# Modeling tooth enamel in FEA comparisons of skulls: Comparing common simplifications with biologically realistic models

**DOI:** 10.1016/j.isci.2021.103182

**Published:** 2021-09-28

**Authors:** Eva C. Herbst, Stephan Lautenschlager, Dylan Bastiaans, Feiko Miedema, Torsten M. Scheyer

**Affiliations:** 1 Palaeontological Institute and Museum, University of Zurich, 8006 Zurich, Switzerland; 2 School of Geography, Earth and Environmental Sciences, University of Birmingham, B15 2TT Birmingham, UK; 3 Staatliches Museum für Naturkunde Stuttgart, 70191 Stuttgart, Germany

**Keywords:** paleontology, biophysics, biomechanics, biomaterials

## Abstract

Palaeontologists often use finite element analyses, in which forces propagate through objects with specific material properties, to investigate feeding biomechanics. Teeth are usually modeled with uniform properties (all bone or all enamel). In reality, most teeth are composed of pulp, dentine, and enamel. We tested how simplified teeth compare to more realistic models using mandible models of three reptiles. For each, we created models representing enamel thicknesses found in extant taxa, as well as simplified models (bone, dentine or enamel). Our results suggest that general comparisons of stress distribution among distantly related taxa do not require representation of dental tissues, as there was no noticeable effect on heatmap representations of stress. However, we find that representation of dental tissues impacts bite force estimates, although magnitude of these effects may differ depending on constraints. Thus, as others have shown, the detail necessary in a biomechanical model relates to the questions being examined.

## Highlights

• We modeled teeth with different properties to test FEA model simplifications
• Changing material properties of teeth did not affect general stress plots
• There are some local stress differences near the constrained teeth between models

## Graphical abstract

**Figure:**
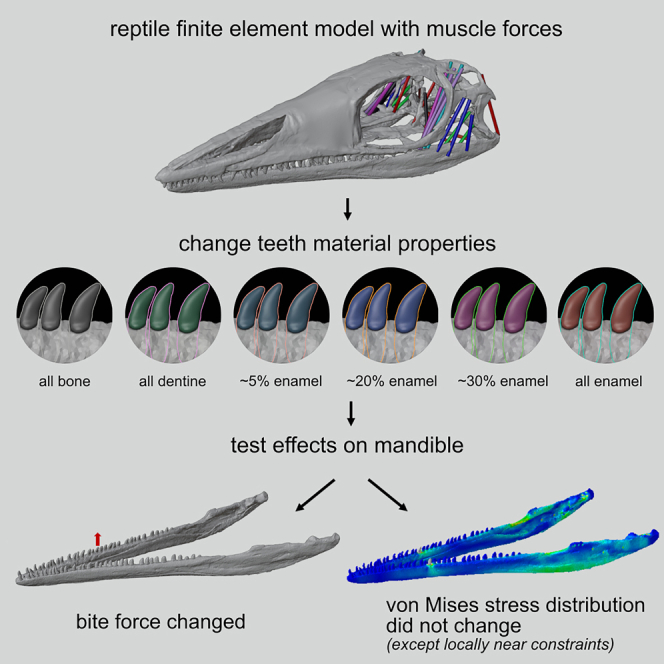

## Introduction

Finite element analysis (FEA) is becoming an increasingly common modeling tool for biologists and palaeontologists to investigate how form relates to function in extinct and extant taxa (Bright 2014). In FEA studies, forces are applied to models which are subdivided into a finite number of elements, to investigate an object's reaction to outer loads. The mesh is usually created from CT scans or a photogrammetry surface mesh and captures the anatomy of the structure of interest. For example, muscle forces can be applied to a skull model to investigate bite force at different teeth, and compare how stresses distribute through different skull shapes in fossils (Lautenschlager et al., 2016, Wilken et al., 2020, Cost et al., 2020; see Bright 2014 for an overview of paleontological applications of FEA). To create a realistic model, the correct material properties need to be assigned to the mesh. These material properties include the Poisson ratio (which describes the relationship between transverse strain to axial strain) and the Young's modulus (the relationship of stress to strain: a measure of the stiffness of a material). Values for these properties are obtained from material property tests on the tissues of extant animals (e.g., Creech 2004; Gilmore et al., 1969; Huang et al., 2005; Karimi et al., 2019; Lee et al., 2000; Lautenschlager et al., 2018; Rees and Hammadeh 2004; Zapata et al., 2010). However, appropriate specimens may not be readily available for testing.

When creating models, researchers often conduct sensitivity studies. Sensitivity studies are integral to developing FEA models because they demonstrate which model simplifications can be made reasonably without affecting the results of the analysis. For example, researchers have tested how including sutures and the chondrocranium, altering muscle parameters and geometry, changing joint and bone material properties, including anisotropy, and including both cortical and trabecular bone affects finite element models (Curtis et al., 2008; Bright and Rayfield 2011; Porro et al., 2011; Tseng et al., 2011; Gröning et al., 2013; Cuff et al., 2015; Jones et al., 2017; Taylor et al., 2017; Wilken et al., 2019).

In skull and mandible finite element (FE) studies (e.g., feeding biomechanics), researchers have to decide what material properties to assign to the teeth. Teeth are composed of dentine with an enamel cap in most taxa. The material properties of bone and dentine are similar, so bone material properties can be used to approximately represent dentine behavior (Fitton et al., 2015; Ashman and Van Buskirk, 1987; Dechow et al., 1993; Kinney et al., 2003). However, enamel has a higher Young's modulus than bone and dentine, which means that it is a stiffer material (Cuy et al., 2002). Single human teeth have been modeled with dentine, enamel, the pulp cavity, and even the periodontal ligament (Karimi et al., 2019). However, such detail is very time-consuming to model for multiple teeth. Although the dentine enamel junction has been successfully detected in extant animals and even some fossils (Sander 1999; Skinner et al., 2008; Jones et al., 2018), this junction is often not visible in CT data, especially in fossils. Therefore, most feeding biomechanics FE analyses model the teeth with all bone material properties, because dentine has similar material properties (Wilken et al., 2020; Cost et al., 2020), or all enamel (Lautenschlager et al., 2016; Taylor et al., 2017). Modeling the tooth as all bone material might underestimate the strength of the tooth, but saves the researchers a lot of time in segmenting the teeth (and in some scans or photogrammetry models the tooth root may not be visible). Modeling the teeth as all enamel might cause unrealistic mechanical failure of the teeth, due to increased brittleness. For example, Reichel (2010) observed areas exceeding the maximum enamel compressive stress (indicating failure) in the all-enamel teeth of their *Stegosaurus* model and concluded that this might be because of the simplification of modeling all teeth with enamel material properties and suggested future studies should test the effects of modeling the tooth with both dentine and enamel layers.

Fitton et al. (2015) compared two simplifications (teeth as all bone or all enamel) and found that modeling teeth with bone or enamel material properties affected the strains in the craniofacial skeleton, especially close to the teeth. Taylor et al. (2017) also tested the effects of material properties of teeth by increasing and decreasing the elastic modulus by 10% from their baseline enamel property values (teeth were modeled as all enamel). However, to our knowledge, no sensitivity study has compared realistic amounts of enamel with models in which the tooth is modeled as all bone or all enamel in reptiles, Tseng et al. 2011 conducted a sensitivity study testing the effects of changing the number of material properties in the mandibles of wolves. In most extant taxa, relative enamel thickness (average enamel thickness divided by the cube root of the volume of dentine) is between 2 and 40 percent of the tooth (Sellers et al., 2019). Enamel thickness is dependent on the interplay of various factors including enamel microstructure, overall tooth shape, diet, ontogeny, composition, and presence of surface topology and position within the dentigerous elements (Sander 1999). Primates have especially high relative enamel thickness, whereas reptiles generally have lower relative enamel thickness (Sellers et al., 2019). Even the thickest enamel layers of highly derived durophagous marine taxa (e.g., ichthyosaurians, mosasaurs, and placodonts) comprise only about 10% of the diameter of the tooth (Sander, 1999). An exception to this is the herbivorous lepidosaur *Eilenodon*, where the enamel volume is about 47% of the overall tooth volume (Jones et al., 2018).

**Figure 1:**
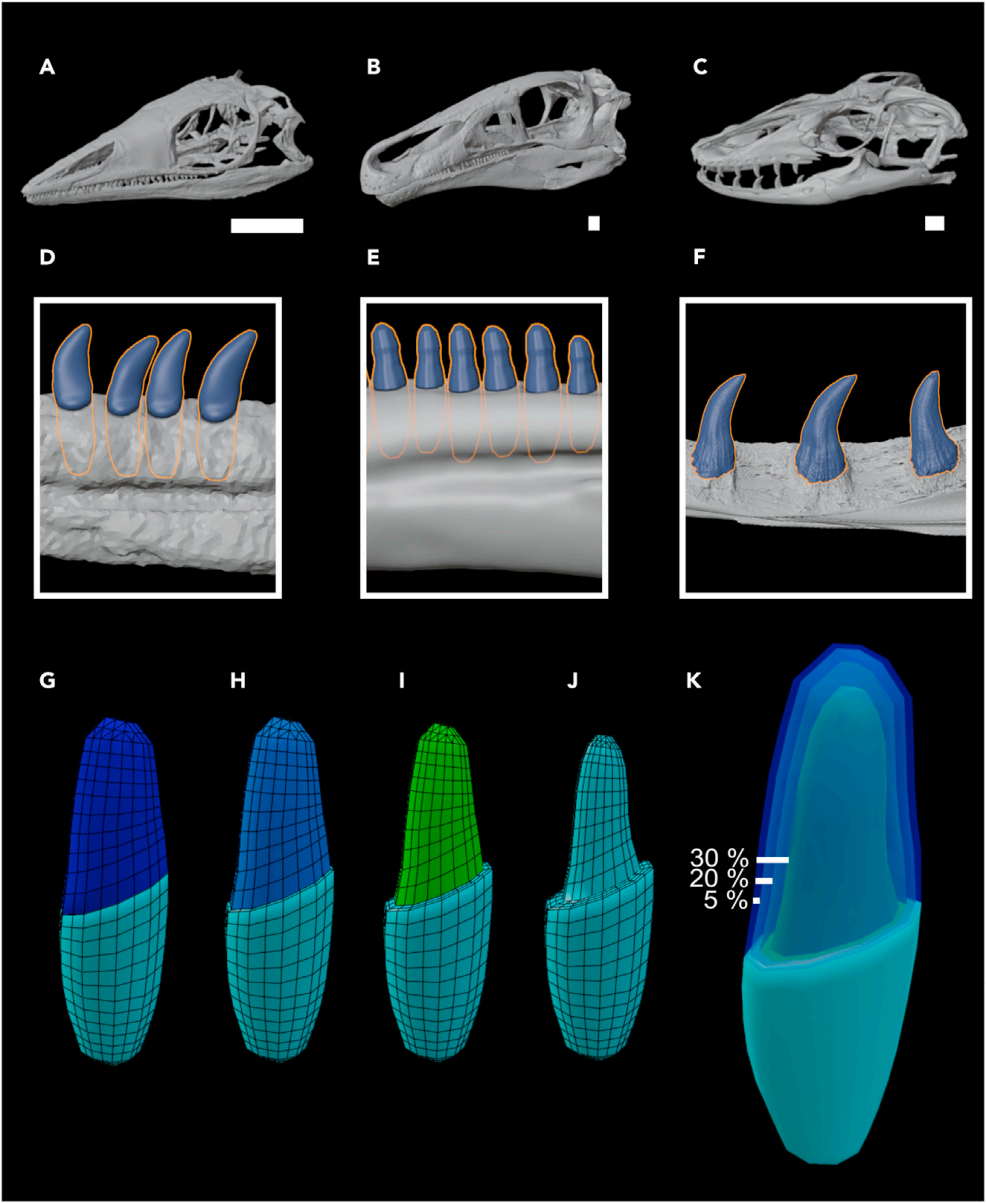
Modeling teeth in extinct and extant reptiles Skull models and detailed views of the mandibular teeth of *Macrocnemus* (A,D), *Erlikosaurus* (B,E), and *Varanus* (C,F). (G–K) Layers created in Blender to model different enamel thicknesses (K). Scale bars = 1 cm.

Here, we test how sensitive FE results are to modeling teeth with biologically realistic amounts of enamel versus modeling teeth with a single material property (either bone, dentine, or enamel). We created lower jaw models from CT scans of the extinct archosauromorph *Macrocnemus bassanii* and herbivorous dinosaur *Erlikosaurus andrewsi*, and the extant monitor lizard *Varanus salvator* (Figures 1A–1C). These reptiles vary in the morphology of their mandibles and teeth and the size of the teeth relative to the mandibles. The ratio of tooth height (measured in the longest dimension at a middle tooth) to mandible length is 3.9% in *Macrocnemus*, 4.5% in *Erlikosaurus*, and 8.1% in *Varanus.* The ratio of tooth height to mandible height (measured at the location of the middle tooth) is 79.6% in *Macrocnemus*, 66.3% in *Erlikosaurus*, and 133.5% in *Varanus.*

The enamel-dentine boundary was not visible in any of the scans. Therefore, we tested a range of enamel thicknesses (∼5–10%, ∼20%, and ∼30% of a cross-section of a middle tooth) (Figures 1G–1K). The upper end of the range of enamel thickness (∼30–40%) is found in extant primates but most reptiles, including dinosaurs, have an enamel thickness of less than 10% (Sellers et al., 2017; Sander 1999). *Varanus salvator* has an enamel layer thickness of 0.02mm (Sander 1999); that study did not report the tooth size but given a tooth anteroposterior length of about 1.7mm at mid-crown in our model, this would be an enamel thickness of <3% in cross-section. The herbivorous therizinosaurid dinosaur *Erlikosaurus andrewsi* has straight blunt leaf-shaped teeth that are constricted at the base. The enamel thickness of other therizinosaurids has been histologically determined in *Falcarius utahensis* (enamel = 2.58% total crown volume) and *Suzhousaurus megatherioides* (enamel = 5.3% total crown volume) (Button et al., 2017). The archosaurs *Revueltosaurus* and *Krzyzanowskisaurus* also have an enamel thickness of less than 10% in cross-section at mid crown (figures 4 and 6 in Heckert and Miller-Camp, 2013). The archosauromorph *Macrocnemus bassanii* from the Triassic of Europe has slightly recurved conical homodont marginal dentition lacking labiolingual compression and having unstriated enamel, whereas having somewhat more bulbous teeth in the palatal region (Jiang et al., 2011; Miedema et al., 2020). *Macrocnemus* is here regarded as having had a diet likely consisting of insects or being more generally carnivorous (Rieppel and Scheffold, 2019) and thus likely having had a thin enamel layer, since in the genus *Varanus* the species feeding on insects and vertebrates have thinner enamel than the durophagous species (Sander 1999). Therefore, our ∼5% enamel scenario is the most realistic for the reptiles tested here. However, we still included a thick enamel layer scenario (∼30%) because even though it is unlikely that these specific extinct reptiles had that much enamel, we wanted to test how a thicker layer of enamel influences the difference between the simplified models versus the models with a layer of enamel, because at least one fossil reptile (*Eilenodon)* has an enamel thickness of over 40% volumetrically (although note that *Eilenodon* has much more bulbous teeth and likely a different diet than the reptiles in this study). The values are approximate because it was difficult to model the exact percentage of enamel (see STAR Methods).

We hypothesize that changing the material properties will not greatly affect the overall patterns of stress distributions in the mandibles, both in the overall patterns of stress distribution (von Mises stress heatmaps) and local von Mises stress at specific nodes along the mandible. Our hypotheses can be explained by physical principles. Following Saint-Venant's principle, stresses converge in regions at a sufficiently large distance from the constraints and load points (Saint-Venant, 1855). This means that regardless of load distribution, regions at a larger distance will show the same stress distribution and may therefore make the effect of the material composition at the bite point negligible. However, it is firstly unclear what the critical distances are for Saint-Venant's principle to take effect, and secondly, biomechanical studies in palaeontology need to consider the complete morphology of a specimen. Furthermore, Saint-Venant's principle becomes less relevant with increasing model complexity, where the critical distance can vary based on the shape and anisotropy of an object (Toupin 1965; Sandorff 1980). Therefore, although we expect the stresses to dissipate further away from the loads, the exact “sphere of influence” of the constraints and load points can only be investigated via modeling in this study.

We further hypothesize that bite forces will differ between the realistic models and the simplified models by a small amount (e.g., less than an order of magnitude), with the all bone model producing the lowest bite force, followed by all dentine, followed by the more realistic amounts of enamel and the all enamel model producing the highest bite force. In scenarios with constant input force (which is true for our models), one might expect the stress to be the same between our different models, and the strain to change. However, at the constraints (e.g., the tooth constraints on the biting tooth) the displacement is fixed (e.g., constant strain) at these nodes. With a change in material properties, we expect the stress to differ in these regions.

## Results

### Estimated bite force

**Figure 2:**
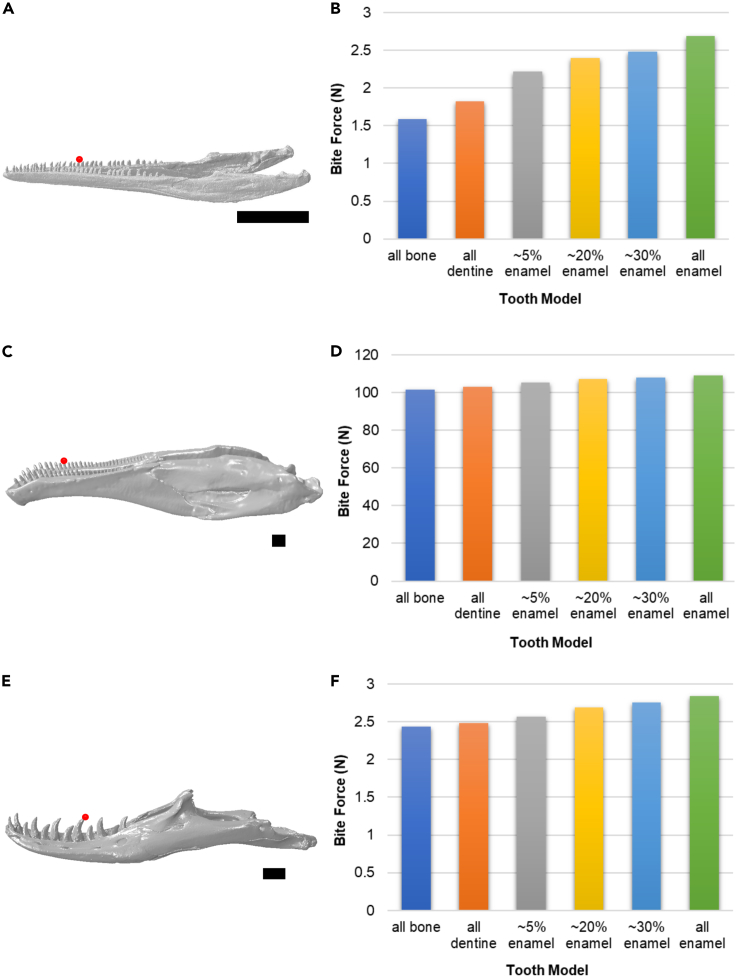
The effect of tooth material properties on bite force The effect of tooth material properties on bite force magnitude in *Macrocnemus* (A, B), *Erlikosaurus* (C,D), and *Varanus* (E,F). Bite forces (in newtons) were measured at a middle tooth on the right side (denoted in red in A,C,E) and the results of the different tooth models are shown in B, D, and F. Scale bars = 1 cm. See also Table S1.

**Figure 3:**
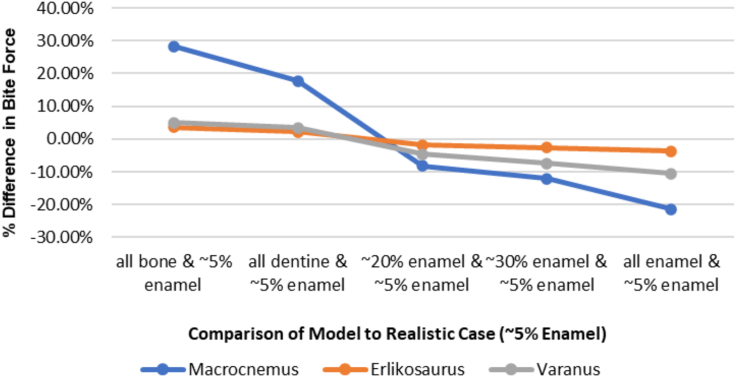
Relative percent difference in bite forces magnitude between all tooth models and the more accurate model (∼5% enamel), calculated as (BiteForce_5% enamel_ - BiteForce_tooth model_)/BiteForce_5% enamel_ See also Table S1.

Our results show that among the evaluated metrics, bite force magnitudes were affected the most by the variation in tooth material properties. The models from least to greatest bite force were as follows: all bone, all dentine, ∼5% enamel, ∼20% enamel, ∼30% enamel, and all enamel (Figure 2). Nodes and bite force values are reported in Table S1. Differences in bite force between model simplifications and the most realistic model (∼5% enamel) ranged from ca. −21% to ca. 28% (Table 1, Figure 3), but without a consistent variation across the different taxa. The bite force differences between models were most pronounced in *Macrocnemus* and least pronounced in *Erlikosaurus.*

**Table 1:** Percent difference in bite force between simplified tooth models and the more accurate model (∼5% enamel), calculated as (BiteForce_5% enamel_ - BiteForce_simplified model_)/BiteForce_5% enamel_

	Difference all bone and 5% enamel	Difference all dentine and 5% enamel	Difference all enamel and 5% enamel
*Macrocnemus*	28.24%	17.76%	−21.41%
*Erlikosaurus*	3.52%	2.23%	−3.66%
*Varanus*	5.06%	3.50%	−10.51%

See also Table S1.

### Contour plots (von Mises stress patterns)

**Figure 4:**
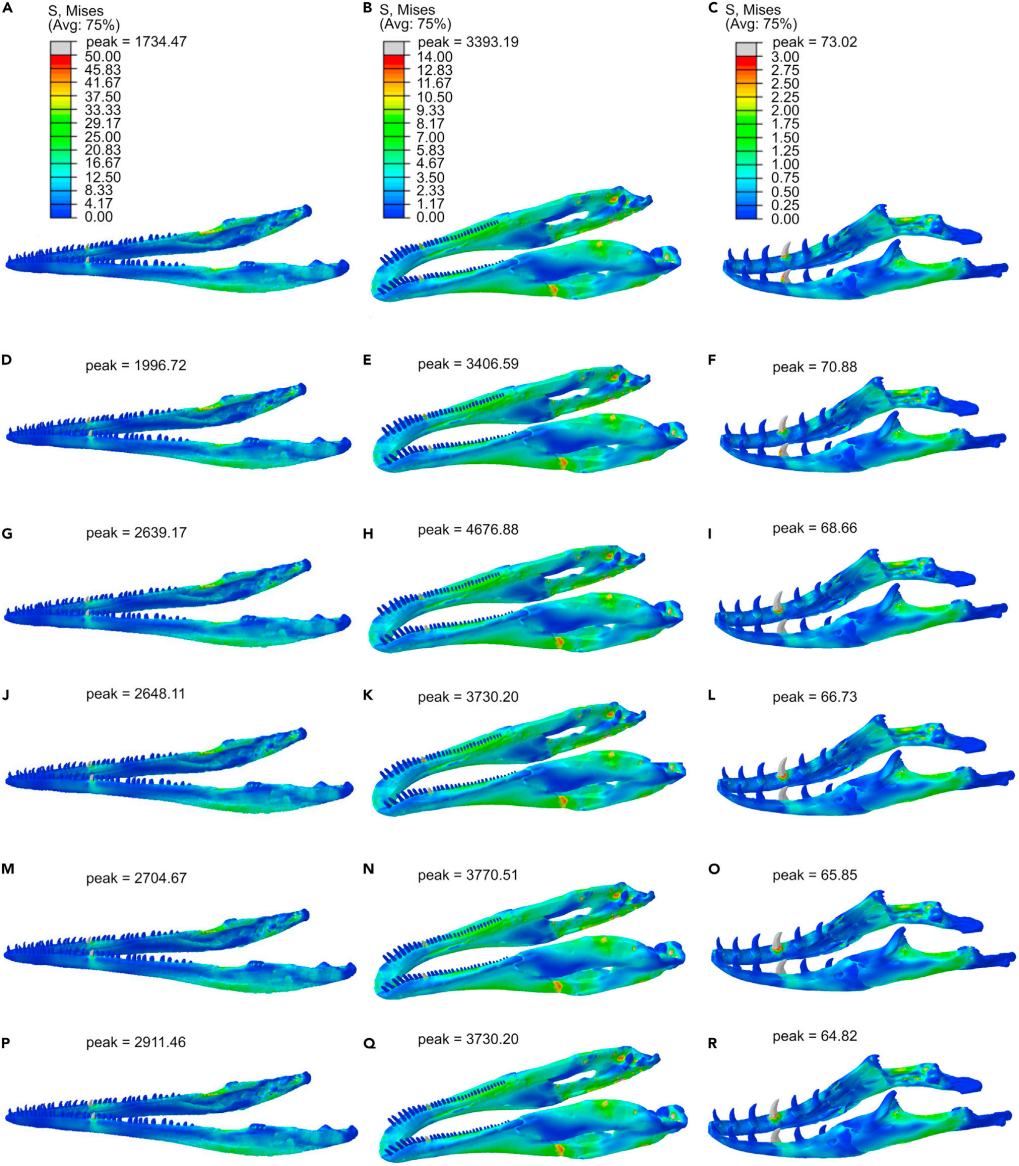
Heat maps of von Mises stress distributions Heat maps of von Mises stress distributions in the mandibles of *Macrocnemus* (A, D, G, J, M, P), *Erlikosaurus* (B, E, H, K, N, Q), and *Varanus* (C, F, I, L, O, R). Teeth were modeled as all bone (A–C), all dentine (D–F), ∼5% enamel (G–I), ∼20% enamel (J–L), ∼30% enamel (M–O), and all enamel (P–R). Teeth were constrained bilaterally. Heatmaps for each model are the same ranges within each species (shown at top) except for peak stresses, which are shown for each individual model. See also Figure S1.

The material properties did not have a visible impact on patterns of von Mises stress distribution across the mandible (Figure 4).

### von Mises stress values

**Figure 5:**
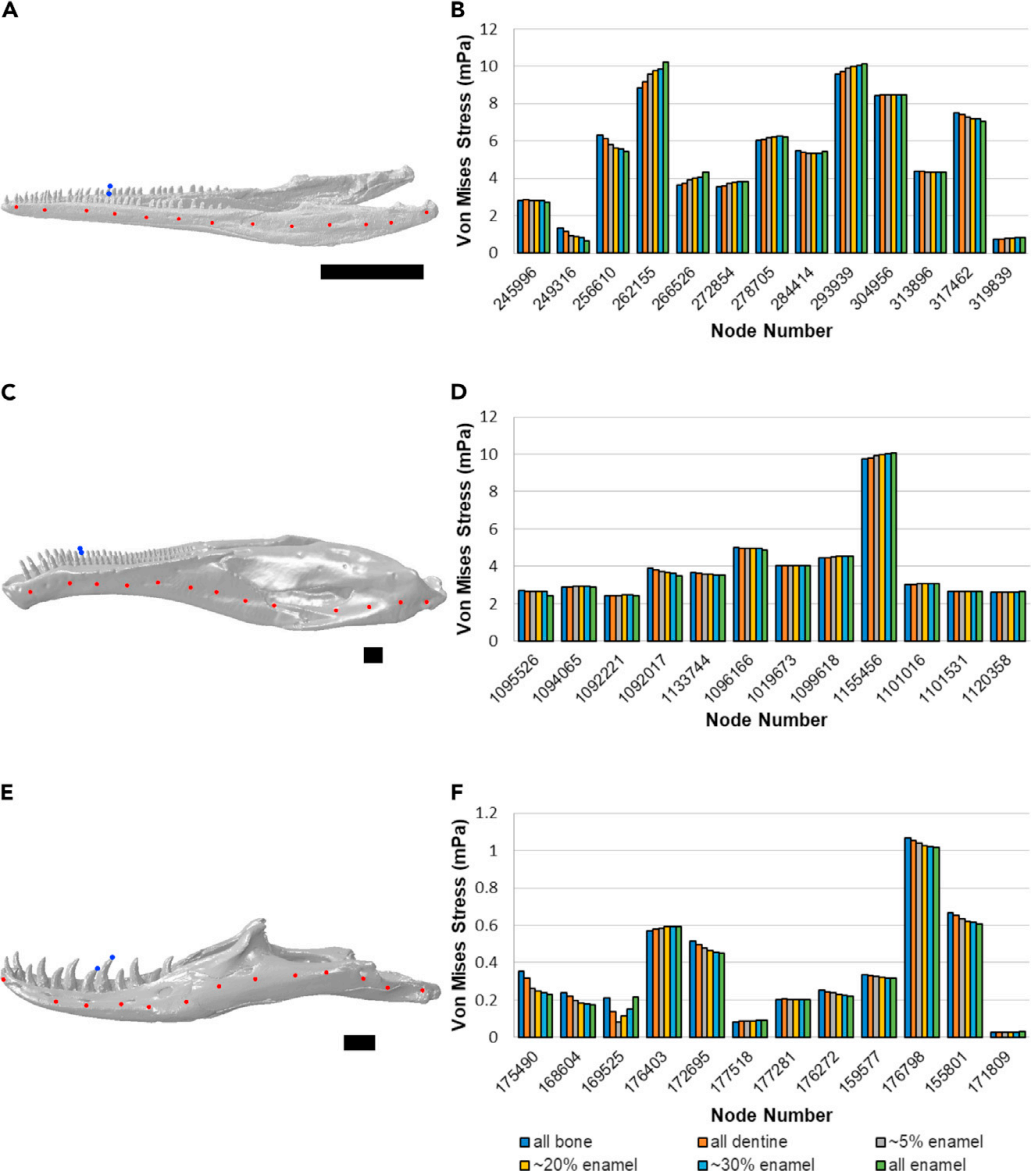
The effect of tooth material properties on absolute von Mises stress value The effect of tooth material properties on absolute von Mises stress values at 13 points along the mandible in *Macrocnemus* (A and B), *Erlikosaurus* (C and D), and *Varanus* (E and F). Sampled points are marked in red, blue points indicate tooth constraints. (A, C, E) Mandible models with the location of queried nodes. (B, D, F) von Mises stress values of specific nodes (anterior-most at left of charts, posterior-most at right). Scale bars = 1 cm. See also Tables S2–S4.

Absolute von Mises stress values at 13 nodes along the mandible were generally similar between the different tooth material property models, with greater absolute differences at specific nodes near the bite points, for example node 169,525 (third-most anterior node) in *Varanus* and node 262,155 (fourth-most anterior) in *Macrocnemus* (Figure 5 and 6; Tables S2–S4).

## Discussion

### Implications for fossil reconstructions

As demonstrated above, the tooth material properties impact absolute bite force values the most (Figures 2 and 3; Table 1; Table S1). Our results support our hypothesis that the all bone model had the lowest bite force, followed by the all dentine model, and the highest bite force was produced by the model with the most enamel (Figure 1; Table S1). We recommend that for studies whose conclusions depend on absolute bite force values, the tooth structure should be modeled in more detail (if the enamel layer is not preserved in a fossil, our Blender tooth layer reconstruction (see STAR Methods) can be used to reconstruct enamel layers if general thickness can be estimated for that taxon). Previous studies highlighted the sensitivity of bite force to other parameters, many of which are uncertain in fossils. For example, Curtis et al. (2008) demonstrated that modeling muscles as straight line segments versus a more realistic muscle wrapping path influences bite force. Absolute bite force values should therefore always be interpreted with caution, because simplifications in the modeling approach can have large impacts.

For the reptiles presented in this study, we would assume that the most accurate enamel thickness is ∼5%. Comparing the bite forces of the simplified models with the ∼5% enamel model gives the error of the simplified model. Differences between all models and the more realistic ∼5% enamel models are given in Table 1 and Figure 3. The error between the other models and the more realistic model is greatest in *Macrocnemus* and less in *Varanus* and *Erlikosaurus*. Furthermore, in *Varanus*, the all bone model and ∼5% enamel model were more similar than the all enamel model and the 5% enamel model. In *Macrocnemus*, the opposite was true and in *Erlikosaurus*, the all bone model and all enamel model were very similar (Figure 3). Models differed in the number of nodes constrained at the jaw joint (see STAR Methods), but we do not think this explains the above differences. To test this, we reduced the number of nodes at the jaw joint constraint in the *Macrocnemus* ∼ 20 percent enamel model from 5 to 4 per side. The overall stress distribution remained the same, the bite force changed very slightly from 2.39714 to 2.39475 N, and the stresses at the nodes only changed very minorly, most noticeable near the jaw joint (less than 0.4% in all points except the posterior-most, which changed by 8%). Instead, the difference in effects of material properties between the different reptiles might be due to differences in morphology, since *Macrocnemus* has the smallest ratio of tooth height to mandible length and *Erlikosaurus* the largest, or differences in muscle forces relative to skull size and morphology. Therefore, the percent difference in bite force between simplified models cannot be generalized for models with different morphology. However, in all taxa, the all dentine model behaved the most similar to the 5% enamel model out of all of the homogeneous simplified models (e.g. the all bone, all dentine, or all enamel models), which is to be expected since the 5% enamel model is mostly dentine. Future studies could build on our data (Table 1) and investigate a larger range of morphologies to determine the general error range in bite forces between simplifications and more realistic models for different morphologies (which could be used as a correction factor for future models).

It is important to note here that for this specific study, bite forces were obtained without any correction factors to account for uncertainty in muscle pennation, and for *Varanus* and *Macrocnemus* the muscle volumes were not based on 3D muscles but instead estimated from the attachment areas. The absolute bite forces and stress shown in this study therefore should be viewed with caution. Several previous FEA studies have investigated bite force and feeding stress in lizards. Dutel et al. (2021) measured an *in vivo* bite force of 211 and 314 N and *ex vivo* bite force of 213 and 308 N in anterior bilateral and posterior unilateral bites of *Salvator merianae* (skull length = 81mm). In *Varanus niloticus* (skull length = 80mm), the *in vivo* bite force was 212 N and 245 N and *ex vivo* bite force was 202 and 252 N in anterior bilateral and posterior unilateral bites, respectively. Bite force reported for *V. komodoensis* differs between different studies. For example, Fry et al. (2009) estimated the bite force of *V. komodoensis* to be 39 N, whereas Moreno et al., 2008 found an axial reaction force at the biting tooth of ∼4 N in mesial and ∼9 N in distal biting at each tooth. Moreno et al., 2008 report the skull length of *V. komodoensis* to be 142mm, which is about the same size as our *Varanus salvator* specimen (which was 148mm). Our results for the bite force of *Varanus salvator* is of similar magnitude as *V. komodoensis* in the Moreno study, lower by about an order of magnitude than the results from Fry et al. (2009) for *S. komodoensis,* and much lower (about 2 orders of magnitude) than the *in vivo* and *ex vivo* reported for *S. merianae* and *V. niloticus.* Rieppel and Labhardt (1979) concluded that *V. niloticus* would have a stronger bite than *V. salvator;* interspecies variation could explain some of the variation between our results and other studies, although the difference in orders of magnitude may also indicate that we underestimated the muscle for in our *V. salvator* model. This could also explain why our stress in *V. salavator* is so much smaller than in our other specimens. However, the general magnitude of stress in our stress plots was similar to the mean stress values reported in Moreno et al., 2008. Fry et al. (2009) also note that differences in the bite force between their models and Moreno et al.’s models could be due to differences in gape angle, which could also explain the differences between our model and the other published studies. Regardless, these differences are not an issue for our study because this research focused on the relative differences between tooth model types, not absolute forces, since we compared each species to different tooth models of itself (not to other species), to test the relative effects of different tooth material properties.

Future studies will conduct a more detailed analysis of the *Macrocnemus* musculature and feeding biomechanics. It is also important to note that our models had approximately 5%, 20%, and 30% enamel, but differed slightly in the exact amounts (see STAR Methods for specific values for each model). However, this does not affect our conclusions because we wanted to test 3 different general ranges of relative enamel thickness (thin, medium, and thick enamel).

Owing to the uncertainties in absolute magnitudes of bite forces, palaeontologists can investigate relative differences between taxa in the stress distributions throughout the skull and mandible (Bright 2014; Lautenschlager et al., 2016; Wilken et al., 2020; Cost et al., 2020). Our study demonstrates that simplifying teeth as either all bone, all dentine, or all enamel does not visibly impact the general stress distribution (e.g., von Mises heatmap) in the mandibles of the reptiles *Macrocnemus*, *Erlikosaurus*, and *Varanus* (Figure 4). Furthermore, von Mises stresses sampled at specific nodes along the mandible were generally very similar between models (Figure 4; Tables S2–S4).

Some nodes, however, did show larger differences in von Mises stress between models. The largest absolute differences in von Mises stress were at node 169,525 (third-most anterior node) in *Varanus* and node 262,155 (fourth-most anterior) in *Macrocnemus* (Figure 5 and 6; Tables S2–S4). These exceptions can be explained by the close location of these points to the tooth bite point constraint (see Figure 2 for tooth constraint and Figure 5 for location of points along the mandible); node 169,525 (third-most anterior node) in *Varanus* and node 262,155 (fourth-most anterior) in *Macrocnemus* are the nodes closest to the bite point constraint. At the tooth constraints, the model has a fixed displacement, which means that the strain at the constraints does not change between the models. Because the strain is constant and material properties changed between the models, this means that at and very close to the constraints, the stress will be different. According to Saint-Venant's principle, far away from the tooth bite constraint we would expect the models to behave similarly. This is demonstrated by our data – the stress values of the nodes closest to the constraints differ the most between models. The local stress distribution very close to the constraints differs locally between models and is shown in Figure S1.

Studies using the tooth model simplifications should therefore sample a range of points across the mandible for comparisons of stress values between taxa, and avoid choosing points too close to constraints (i.e., bite point constraints, jaw joint constraints, or muscles).

Although we used two different approaches to model the enamel (and the *Erlikosaurus* and *Macrocnemus* Blender models ended up having slightly higher enamel for the ∼5% enamel thickness), we do not think that these differences had a big effect on the outcomes. Indeed, the *Varanus* model (with tooth layers created in Avizo) was more similar to the *Erlikosaurus* model (with tooth layers created in Blender) in terms of the relationship of bite force in simplified models compared to the ∼5% enamel model (Table 1) than either were to the *Macrocnemus* model (with tooth layers created in Blender).

Overall, our study demonstrates that for biomechanical studies in reptiles with a range of mandibular and tooth morphologies, comparing general stress distributions between different taxa, simplifying teeth as all bone, all dentine, or all enamel is reasonable. However, the effect of material properties on stress profiles also likely depends on the size of the teeth relative to the mandible. For example, Tseng et al. (2011) found that changing the number of material properties in mandible models of wolves changed the stress distributions. Mapping stress contour plots is commonly used in comparative biomechanical studies (Lautenschlager et al., 2016; Wilken et al., 2020; Cost et al., 2020), or in hypothetical deductive models that investigate the effects of the addition or removal of specific anatomical features to test their function (Rayfield, 2007; Moazen et al., 2009; Anderson et al., 2011; Bright 2014). Using all bone material properties can save the researcher a lot of time segmenting out the teeth. Furthermore, the tooth roots might not be visible in some scans, preventing their segmentation. However, if the results depend on absolute bite force, the tooth structure should be modeled in more detail.

This influence of tooth material properties on bite force was also observed by Tseng et al. (2011) in wolf mandibles. It is important to note that in both of their and our study, the joint reaction force decreased when the bite force increased and vice versa. In other words, the distribution of forces changes, but the total output force stays the same. This is necessary to achieve equilibrium in the model, i.e. input force must equal output force, and since we did not change input forces between models, the net output force must stay consistent between models. The same applies to moments. We double checked out model and we confirmed that between models, net output forces and moments stayed consistent.

### Limitations of the study

A limitation of our study is that we did not model the periodontal ligament in *Erlikosaurus* and *Macrocnemus.* Periodontal ligaments are present in mammals and crocodiles, whereas in lepidosaurs teeth are usually ankylosed to the mandible without a ligamentous attachment (Maxwell et al., 2011; Porro et al., 2011; Bertin et al., 2018). In *Varanus*, the teeth are ankylosed to the jaw, but *Erlikosaurus* and *Macrocnemus* might have had periodontal ligaments. The teeth of *Macrocnemus* were probably sub-thecodont (Renesto and Avanzini 2002; Jaquier et al., 2017). The reason we excluded the periodontal ligament is that paleontological studies rarely model it, likely due to the increased time needed to create such models. Furthermore, material properties for the periodontal ligaments are unknown. Even for extant taxa such as crocodylians, the material properties have not been tested. Porro et al. (2011) modeled the periodontal ligament in alligator mandibles, using material properties in between mammalian periodontal ligaments and the mineralized connection found in lizards. There has been debate over the extent to which the periodontal ligament (PDL) influences finite element analyses (Wood et al., 2011; Gröning et al., 2011; Marinescu et al., 2005; Gröning and Fagan 2012). These studies were conducted on primate skulls and mandibles, and the discrepancies in the results may be due to the different regions (skull versus mandible) that were analyzed. Previous studies have shown that including sutures can affect the FEA strain distributions in specific regions (Wang et al., 2010; Jones et al., 2017), and perhaps including sutures in the Wood et al., (2011) model may have produced larger differences between the models with and without the PDL, since modeling sutures generally increases strain and can change strain distribution in certain regions. On the other hand, the differences may simply be because of shape differences in the mandible and skull. To our knowledge, Porro et al. (2011) is the only study that tested the effects of the PDL in an FEA study of reptiles. Porro et al. (2011) found that adding the PDL as well as sutures and anisotropy to the model decreased bite forces and reduced dorsoventral bending, maximum torsional, and maximum shear stresses. However, these effects are due to the combination of the more accurate model with PDL, sutures, and anisotropy. Future studies could investigate the effects of the PDL in *Macrocnemus* and *Erlikosaurus.*

In *Varanus*, future studies could model the tooth attachment in more detail by including the cementum layer, which attaches the tooth to the jawbone (Maxwell et al., 2011). Cementum has a slightly lower elastic modulus than dentine, based on indentation studies in rat molars (Ho et al., 2009). Future studies could investigate how the periodontal ligament and cementum influence the effects of tooth material property simplifications.

Another limitation of our study is that we only investigated reptiles and a limited amount of tooth morphologies. We expect that for mammals, and in particular primates, which generally have much thicker enamel layers, the all enamel simplification would produce more realistic results than the all-bone simplification. However, the shape and size of the teeth relative to the mandible likely also influence the sensitivity of the model to tooth material properties; in wolf mandibles, the stress distribution changes more in the different model variations than in our study (Tseng et al., 2011).

We also used linear measurements of percent enamel thickness (in tooth cross-section) because this was easier to implement – some studies (e.g. Sander 1999) report linear thickness of the enamel layer, whereas other studies measure relative enamel volumetrically (e.g., Sellers et al., 2017). Although 5% linear enamel thickness would not be exactly the same as 5% volumetric enamel thickness, these values are close enough for our study since we just tested approximate values corresponding to “thin” (= realistic for these reptiles), “medium”, and “thick” enamel. Furthermore, for many of the specimens the resolution of scans and lack of histological data makes it impossible to detail specific enamel distributions. In the absence of obvious surface topology we assume that enamel distribution is generally even although in reality the enamel distribution can vary, decreasing slightly from tip of the crown to the base in most reptiles (Rieppel and Labhardt 1979; Sander 1999; Heckert and Miller-Camp, 2013; García and Zurriaguz, 2016; Jones et al., 2018).

We recommend further sensitivity studies for mammals modeling a wider range of tooth shapes and sizes, as well as other reptiles such as *Eilenodon* (which has bulbous teeth and very thick enamel), including tests of the effect of muscle forces, to determine general error ranges and correction factors if the researchers are interested in magnitudes of errors in stress distribution and bite forces introduced by simplifying the material properties.

Another limitation of our study is that we completely constrained the teeth, which might influence the magnitude of the effect of material properties on bite force. When we tested the components of the bite forces, we found the most variation between models in the global Y direction (the anteroposterior direction), instead of the dorsoventral direction (the expected primary direction of the biting action). While we expect the curved teeth to have some posterior component, this was also the case in the *Erlikosaurus* model with straighter teeth. The high Y force component variation could be due to the constraints preventing motion in the Y axis, and could be an explanation of why our models show such a large change with a change in material properties. Constraining teeth in all axes is not uncommon and our results therefore are relevant for showing the effect of material properties on bite force magnitudes given these constraints. However, future studies should test constraining the teeth and jaw joints in fewer axes, to investigate whether this reduces the magnitude of the differences in bite forces between models.

## STAR★Methods

### Key resources table

**Table:** 

REAGENT or RESOURCE	SOURCE	IDENTIFIER
**Deposited data**		

*Macrocnemus bassanii*	data deposited on Morphosource: http://n2t.net/ark:/87602/m4/363412 (*Macrocnemus mandible)* http://n2t.net/ark:/87602/m4/363418*Macrocnemus inner tooth and root)* http://n2t.net/ark:/87602/m4/363497*(Macrocnemus thick enamel layer)* http://n2t.net/ark:/87602/m4/363519*(Macrocnemus middle tooth layer)* http://n2t.net/ark:/87602/m4/363532*(Macrocnemus thin enamel layer)*	000363412 (mandible), 000363418 (root and inner tooth), 000363497 (tooth layer), 000363519 (tooth layer), 000363532 (tooth layer)
*Erlikosaurus andrewsi*	data deposited on Morphosource:http://n2t.net/ark:/87602/m4/363549 (*Erlikosaurus mandible)* http://n2t.net/ark:/87602/m4/363557*(Erlikosaurus inner tooth and root)* http://n2t.net/ark:/87602/m4/363561*(Erlikosaurus thick enamel layer)* http://n2t.net/ark:/87602/m4/363565*(Erlikosaurus middle tooth layer)* http://n2t.net/ark:/87602/m4/363569*(Erlikosaurus thin enamel layer)*	000363549 (mandile), 000363557 (inner tooth and dentine), 000363561 (tooth layer), 000363565 (tooth layer), 000363569 (tooth layer)
*Varanus salvator*	data deposited on Morphosource: http://n2t.net/ark:/87602/m4/363397*(Varanus mandible)* http://n2t.net/ark:/87602/m4/363405*(Varanus teeth)* http://n2t.net/ark:/87602/m4/363320*(Varanus mandible and teeth)*	000363397 (mandible), 000363405 (teeth), 000363320 (mandible and teeth)

**Software and algorithms**		

Blender (version 2.91.0)	Blender Foundation, community	RRID:SCR_008606, http://www.blender.org
Hypermesh (version 11)	Altair engineering	https://www.altair.com/hypermesh/
Avizo (version 6.3.1 & 7.0.0)	Thermo Fisher Scientific	RRID:SCR_014431, http://www.fei.com/software/avizo3d/
Abaqus (version 6.14)	Simuleon	https://www.simuleon.com/simulia-abaqus/

### Resource availability

#### Lead contact

Further questions should be directed to the lead contact, Eva C. Herbst (eva.herbst@pim.uzh.ch).

#### Materials availability

This study did not generate new reagents.

### Experimental model and subject details

#### Animals

All specimens were either fossil specimens or extant museum specimens preserved in the collections. Details such as museum numbers are provided in the method details section.

### Method details

**Institutional abbreviations:** Palaeontological Institute and Museum, University of Zurich (PIMUZ); Geological Institute of the Mongolian Academy of Sciences, Ulaan Baatar, Mongolia (IGM)

#### Specimens and segmentation

The *Varanus salvator* specimen (PIMUZ A/III 1493) was scanned at 77.643 microns (Nikon XTH 225 ST) and the mandible and teeth were segmented in Mimics (version 23.0, Materialise NV). The *Macrocemus bassanii* (PIMUZ T 2477) skull and cranium were modified from Miedema et al., (2020) (scanned at ID19 beamline of the European Synchrotron Radiation Facility, voxel size 18.21 microns). We segmented the teeth (Mimics version 23.0, Materialise NV) and retrodeformed the skull using Blender (version 2.91.0, GNU general public license). The cranium was used for muscle attachment areas; the full skull model and more details on retrodeformation will be published in a future paper (Herbst et al. in prep). In all specimens, the right side of the mandible was mirrored across the midline to create the mandible. For *Varanus* we also segmented out the pulp cavity, which was visible in the CT data. *Erlikosaurus* (IGM 100/111) was scanned at 145 microns (XT-H-225ST CT scanner) and the model was segmented in Avizo (version 6.3.1 & 7.0.0, Thermo Fisher Scientific); it was initially created for another study (Lautenschlager 2013). A surface mesh of the mandible was then generated from the sliced model.

#### Modeling enamel layers

We used two approaches for modeling the enamel layers. For *Varanus*, the enamel layers were created in the segmentation editor in Avizo. The *Varanus* surface models were first converted back into slice data in Avizo. We picked a middle tooth on the right side for the measurements, and used planar measurements in the mediolateral cross-section as a proxy for enamel percentage. We then resampled the images so that one pixel would equal 2.5% of the overall tooth cross-section at the widest part of the tooth. This resampling allowed us to create the necessary enamel thickness layers, the thinnest being 5% at the widest part of this tooth (1 pixel on each side). The resampling magnitude was 7/5x for the *Varanus* model. The percentage of enamel in extant animals is often computed based on a volumetric approach, where the relative enamel thickness is the 3D average enamel thickness divided by the cube root of the volume of dentine (Olejniczak et al., 2008; Sellers et al., 2019). Here we used planar measurements of enamel as a proxy for enamel thickness, since the planar measurement approach enabled us to resample to the required resolution right away. Creating volumetric measurements would have required us to create the layers before resampling and then conduct iterative repetitions of resampling, layer creation, and volume calculations, which was not feasible. For the enamel layer creation itself, we used volume growing and shrinking since this is more biologically realistic than growing the enamel layers on a slice by slice basis.

After resampling the images, we created three thicknesses of enamel. We first locked the background and bone and then used a 1-pixel volumetric grow and shrink operation to create a layer that was the outer 5% of the tooth. We then repeated this procedure to create two more layers, one with a 2-pixel volumetric grow and shrink, and the innermost layer with a 3-pixel volumetric grow and shrink. The single outer layer created an enamel layer comprising about 5% of the tooth, two outer layers together created an enamel layer comprising about 20% of the tooth, and all three layers together creating an enamel layer comprising about 30% of the tooth. Because it was impossible to create a pixel size of precisely 2.5% of the tooth volume, the actual percentages varied slightly. For *Varanus*, the cross-sectional enamel thickness relative to the dentine thickness was 5.3% for the thinnest enamel layer, 17.2% for the intermediate enamel layer, and 28.4% for the thickest enamel layer.

However, this image resampling produced a very high-resolution model which had to be downsampled for the creation of the FE models. During the downsampling some of the thin tooth layers intersected each other. These regions were fixed in Hypermesh, ensuring the layer thicknesses were maintained. To avoid these intersections, we developed an alternate modeling method for the teeth of *Erlikosaurus* and *Macrocnemus* using Blender (version 2.91.0, GNU general public license).

First, we removed all teeth and sockets from the model. Using a segmented tooth as a template, we recreated the tooth morphology in Blender in a box-modeling approach (Rahman and Lautenschlager 2016). We then separated the root and crown into two different objects. We duplicated the crown several times and shrank it to create correctly sized enamel caps (Figures 1G–1K).

We again used planar measurements as a proxy for enamel thickness, because this facilitated ease of modeling. We aimed for about 5%, about 20%, and about 30% layers. The *Macrocnemus* thin enamel layer was 8.4%, the medium layer was 19.2%, and the thickest layer was 30%, again measured at a middle tooth at the thickest part of the base of the model. The *Erlikosaurus* enamel layer thicknesses were 8.5%, 19.4%, 33.2%. These measurements varied slightly from the goal measurements because of slight shape adjustments we performed after creating the Blender tooth, to better model the tooth morphology.

To create the different teeth along the mandible, we duplicated and scaled the initial tooth. We also used the lattice modifier in Blender to achieve the slight differences in tooth morphology along the mandible, using the segmented teeth from the CT scan as a reference. The upside to the Blender approach is that when teeth are scaled, the relative sizes for the enamel layers remain correct, whereas in the Avizo approach, a smaller tooth would have relatively more enamel, since the enamel thickness was defined by the number of pixels. This is not a problem for our *Varanus* model since the *Varanus* teeth are fairly uniform in size.

The duplicating and scaling of the tooth layers in Blender enabled us to make independent water-tight meshes for each layer that were flush with each other (e.g. sharing the same node locations on their boundaries). This was important for ensuring that we could assign the layers different material properties but still propagate forces through them. We also duplicated the root to create a socket that was flush with the root (e.g. sharing the same node locations on their boundaries). This Blender method avoided the mesh intersection problems caused by the Avizo approach. In the Avizo approach the whole model got downsampled uniformly (keeping it at the original high resolution would not have been possible computationally), and therefore the thinnest enamel layer had some intersections with the next layer, which needed to be manually corrected in Hypermesh. However, in the Blender approach the teeth are modeled separately from the mandible, and each enamel layer is perfectly nested within the adjacent layers (with the same face geometry), enabling the creation of thin structures without the downsampling issues.

#### Muscle reconstructions

The *Erlikosaurus* muscles were reconstructed three-dimensionally in Avizo based on osteological correlates and comparisons with extant taxa (Lautenschlager 2013.) For the muscle forces, we used the higher muscle forces of the ranges reported in Lautenschlager (2013, Table 1), which were calculated from cross-sectional area in Avizo. Lautenschlager (2013) reported a range of muscle forces values to account for uncertainties in muscle pennation (Thomason 1991). However, here, since we are not interested in the exact bite forces or stress distributions but rather in comparing the effects of different enamel layers, we did not test a range.

For *Varanus*, only the mandibular insertions were used to approximate muscle cross-sectional area for ease and quickness of modeling, since rough estimates of muscle force were sufficient for this study, as we were not interested in precise absolute bite forces and stress values. For *Macrocnemus*, we estimated muscle volumes by calculating the volume of a frustum based on the origin and attachment areas (Sellers et al., 2017). Muscle origins and attachments were inferred using osteological correlates and extant phylogenetic bracketing in the fossil, and we assumed 0 muscle pennation. A more detailed account of our *Macrocnemus* muscle reconstructions, including a correction factor to account for a range of muscle pennations, will be reported in a future study (Herbst et al. in prep). However, for this study simplifications in the muscle anatomy are valid, since we are not interested in absolute bite forces and stress distributions but instead are testing the relative impact of changing the tooth material properties. Muscle physiological cross-sectional area (CSA) was obtained by dividing the muscle volume by the muscle length. The force of each muscle was calculated (Equation 1) assuming an isometric muscle stress (i.e. specific tension) value of 0.3 N mm^-2^ following Thomason (1991), Lautenschlager (2013), and Cost et al., (2020).

(Equation 1) F_mus_ = CSA x muscle stress

All muscles were modeled as simplified cylinders which were created in Blender (1-3 cylinders, depending on the size of the muscle). These muscle cylinders were used to determine the direction of the force vectors in Hypermesh. Muscle wrapping was not included in the models, because we were interested in the comparison of different amounts of enamel rather than in creating the most realistic model. The *Erlikosaurus* muscle cylinders were previously published in Lautenschlager (2015).

#### Finite element analysis

Surface meshes were imported into Hypermesh (v.11, Altair engineering) and tetrameshed. The *Macrocnemus* model had 452,104 nodes and 2,212,962 elements, the *Erlikosaurus* model had 1,184,730 nodes and 6,434,338 elements, and the *Varanus* model had 170,573 nodes 711,481 elements. Bright and Rayfield (2011) showed that in a pig cranium model, four noded tetrahedral element meshes converted to within 5% by 1,750,000 elements. Our *Erlikosaurus* and *Varanus* models were below this, but Tseng et al. (2011, Table S2) demonstrated that the strain energy between 704,257 and 1,404,279 elements differed by only 4.6%. McCurry et al., (2015) showed that reducing the surface mesh resolution 15-fold (but keeping the total number of volumetric elements constant) resulted in a difference of only 10% in von Mises strain. We therefore conclude that our mandible models are sufficiently high resolution for this study, especially since we are not investigating absolute bite forces or stress values but instead investigating relative differences in models with varying amounts of enamel.

For the *Varanus* model, the teeth were imported from Avizo as .hmascii files (which save the boundaries of Avizo materials to automatically connect the components). For *Erlikosaurus* and *Macrocnemus*, we imported separate .stls from Blender into Hypermesh and then used the “node equivalence” function to connect the components (e.g. connecting the nodes on the inside of the enamel layer with the shared nodes on the outside of the dentine layer). This ensures that forces propagate between the different tooth layers but enables the assignment of different material properties to the different layers. For each specimen we created six different FEA models, which varied only in the material properties of the tooth layers. The teeth were assigned either all bone material properties, all dentine, approximately 5% enamel, approximately 20% enamel, approximately 30% enamel, and all enamel. For the increasing amount of enamel, we first assigned only the outer tooth layer to the enamel to obtain about 5% enamel and the rest dentine, then added the middle layer to obtain about 20% enamel, and then added the third layer as enamel to get about 30% enamel. In *Varanus,* the pulp cavity was included in all models, and a mandibular symphysis was modeled with bone material properties to connect the two hemi-mandibles to reduce the complexity of the analysis (in reality, the two hemi-mandibles are not fused). The material properties that we used in all of the model components are shown in Table

**Table:** Material properties used for finite element analysis

Material	Young's modulus (E)	Poisson ratio (v)	Reference
Bone	15,000 MPa	0.29	Porro et al., (2011) (based on average of Zapata et al., 2010 anisotropic values of *Alligator mississippiensis*)
Dentine	21,000 MPa	0.31	Gilmore et al., 1969 (bovine)
Enamel	60,400 MPa	0.30	E: Creech (2004) (*Alligator mississippiensis*) v: Rees and Hammadeh (2004) (human)
Pulp cavity	6.89 MPa	0.45	Karimi et al., (2019), Huang et al., (2005), Lee et al., (2000) (human)

. Palaeontologists use values from extant taxa to approximate the material properties for extinct taxa, and not all relevant material properties have been tested for reptiles, which is why we also included mammalian values for some of the properties.

The jaw joints and middle teeth on both sides (Figure 2) were constrained in all directions in all three model (1 node at each tooth for bite point constraints for all species, 5 nodes per side for *Macrocnemus* jaw joint constraint, 3 nodes at the jaw joint for *Erlikosaurus*, and 4 nodes at the jaw joint of *Varanus*). The number of nodes were based on the morphology of the joint, with more constraints for more anteroposteriorly elongate joints. However, the number of nodes should not matter except very locally (e.g. at the singularity experienced at the constrained node). We confirmed this by doing a sensitivity test (see discussion). Muscle forces were added as load vectors. The model was analysed using Abaqus (v. 6.14, Simuleon). Bite forces were calculated at a middle tooth on the right side (the same tooth that was constrained, see Figure 2). We calculated overall bite force magnitude because in the curved teeth, the axis normal to the tooth does not align with the global dorsoventral axis, so testing only dorsoventral components of the reaction force does not reflect the components in the antero-posterior direction in the curved teeth. Local von Mises stress was calculated for 13 nodes along each mandible (Figure 5).

## Data Availability

• 3D mandible and tooth models have been deposited on MorphoSource.org and are publicly available as of the date of publication. Accession numbers are listed in the key resources table. For the models in which tooth layers were created in Blender (*Macrocnemus, Erlikosaurus)* we archived all tooth layers. Note that all the layers are nested within each other and combined to get the correct amounts of enamel thickness (e.g. to create the thick (∼30 percent) enamel layer, the thick enamel layer *and* the middle and thin layers were assigned enamel material properties). For *Varanus* the tooth surface models intersected after downsampling (see STAR Methods) and were modified after tetrameshing; therefore, we archived the whole tooth without separate layers. • No original code was produced in this study. • Any additional information required to reanalyze the data reported in this paper is available from the lead contact upon request.

## References

[bib1] Anderson P.S., Gill P.G., Rayfield E.J. (2011). Modeling the effects of cingula structure on strain patterns and potential fracture in tooth enamel. J. Morphol..

[bib2] Ashman R.B., Van Buskirk W.C. (1987). The elastic properties of a human mandible. Adv. Dental Res..

[bib3] Bertin T.J., Thivichon-Prince B., LeBlanc A.R., Caldwell M.W., Viriot L. (2018). Current perspectives on tooth implantation, attachment, and replacement in amniota. Front. Physiol..

[bib4] Bright J.A. (2014). A review of paleontological finite element models and their validity. J. Paleontol..

[bib5] Bright J.A., Rayfield E.J. (2011). The response of cranial biomechanical finite element models to variations in mesh density. Anat. Rec..

[bib6] Button K., You H., Kirkland J.I., Zanno L. (2017). Incremental growth of therizinosaurian dental tissues: implications for dietary transitions in Theropoda. PeerJ.

[bib7] Cost I.N., Middleton K.M., Sellers K.C., Echols M.S., Witmer L.M., Davis J.L., Holliday C.M. (2020). Palatal biomechanics and its significance for cranial kinesis in *Tyrannosaurus rex*. Anat. Rec..

[bib8] Creech J. (2004).

[bib9] Cuff A.R., Bright J.A., Rayfield E.J. (2015). Validation experiments on finite element models of an ostrich (*Struthio camelus*) cranium. PeerJ.

[bib10] Curtis N., Kupczik K., O’Higgins P., Moazen M., Fagan M. (2008). Predicting skull loading: applying multibody dynamics analysis to a macaque skull. Anat. Rec..

[bib11] Cuy J.L., Mann A.B., Livi K.J., Teaford M.F., Weihs T.P. (2002). Nanoindentation mapping of the mechanical properties of human molar tooth enamel. Arch. Oral Biol..

[bib12] Dechow P.C., Nail G.A., Schwartz-Dabney C.L., Ashman R.B. (1993). Elastic properties of human supraorbital and mandibular bone. Am. J. Phys. Anthropol..

[bib13] Dutel H., Gröning F., Sharp A.C., Watson P.J., Herrel A., Ross C.F., Jones M.E., Evans S.E., Fagan M.J. (2021). Comparative cranial biomechanics in two lizard species: impact of variation in cranial design. J. Exp. Biol..

[bib14] Fitton L.C., Prôa M., Rowland C., Toro-ibacache V., O’higgins P. (2015). The impact of simplifications on the performance of a finite element model of a *Macaca fascicularis* cranium. Anat. Rec..

[bib15] Fry B.G., Wroe S., Teeuwisse W., van Osch M.J., Moreno K., Ingle J., McHenry C., Ferrara T., Clausen P., Scheib H. (2009). A central role for venom in predation by *Varanus komodoensis* (Komodo Dragon) and the extinct giant *Varanus (Megalania) priscus*. Proc. Natl. Acad. Sci..

[bib16] García R.A., Zurriaguz V. (2016). Histology of teeth and tooth attachment in titanosaurs (Dinosauria; Sauropoda). Cretaceous Res..

[bib17] Gilmore R.S., Pollack R.P., Katz J.L. (1969). Elastic properties of bovine dentine and enamel. Arch. Oral Biol..

[bib18] Gröning F., Fagan M.J. (2012). Comment on “The effects of modelling simplifications on craniofacial finite element models: the alveoli (tooth sockets) and periodontal ligaments” (volume 44, issue 10, pages 1831-1838). J. Biomech..

[bib19] Gröning F., Fagan M.J., O’Higgins P. (2011). The effects of the periodontal ligament on mandibular stiffness: a study combining finite element analysis and geometric morphometrics. J. Biomech..

[bib20] Gröning F., Jones M.E., Curtis N., Herrel A., O’Higgins P., Evans S.E., Fagan M.J. (2013). The importance of accurate muscle modelling for biomechanical analyses: a case study with a lizard skull. J. R. Soc. Interface.

[bib21] Heckert A.B., Miller-Camp J.A. (2013). Tooth enamel microstructure of *Revueltosaurus* and *Krzyzanowskisaurus* (Reptilia: Archosauria) from the upper triassic chinle group, USA: implications for function, growth, and phylogeny. Palaeontol. Electronica.

[bib22] Ho S.P., Yu B., Yun W., Marshall G., Ryder M.I., Marshall S.J. (2009). Structure, chemical composition and mechanical properties of human and rat cementum and its interface with root dentin. Acta Biomater..

[bib23] Huang H.M., Ou K.L., Wang W.N., Chiu W.T., Lin C.T., Lee S.Y. (2005). Dynamic finite element analysis of the human maxillary incisor under impact loading in various directions. J. Endod..

[bib24] Jaquier V.P., Fraser N.C., Furrer H., Scheyer T.M. (2017). Osteology of a new specimen of *Macrocnemus aff. M. fuyuanensis* (Archosauromorpha, Protosauria) from the middle triassic of Europe: potential implications for species recognition and paleogeography of tanystropheid protorosaurs. Front. Earth Sci..

[bib63] Jiang D.Y., Rieppel O., Fraser N.C., Motani R., Hao W.C., Tintori A., Sun Y.L., Sun Z.Y. (2011). New information on the protorosaurian reptile Macrocnemus fuyuanensis Li et al., 2007, from the Middle/Upper Triassic of Yunnan, China. J Vertebr Paleontol.

[bib25] Jones M.E., Gröning F., Dutel H., Sharp A., Fagan M.J., Evans S.E. (2017). The biomechanical role of the chondrocranium and sutures in a lizard cranium. J. R. Soc. Interface.

[bib26] Jones M.E., Lucas P.W., Tucker A.S., Watson A.P., Sertich J.J., Foster J.R., Williams R., Garbe U., Bevitt J.J., Salvemini F. (2018). Neutron scanning reveals unexpected complexity in the enamel thickness of an herbivorous Jurassic reptile. J. R. Soc. Interface.

[bib27] Karimi A., Razaghi R., Biglari H., Rahmati S.M., Sandbothe A., Hasani M. (2019). Finite element modeling of the periodontal ligament under a realistic kinetic loading of the jaw system. Saudi Dental J..

[bib28] Kinney J.H., Marshall S.J., Marshall G.W. (2003). The mechanical properties of human dentin: a critical review and re-evaluation of the dental literature. Crit. Rev. Oral Biol. Med..

[bib29] Lautenschlager S. (2013). Cranial myology and bite force performance of *Erlikosaurus andrewsi*: a novel approach for digital muscle reconstructions. J. Anat..

[bib30] Lautenschlager S. (2015). Estimating cranial musculoskeletal constraints in theropod dinosaurs. R. Soc. Open Sci..

[bib31] Lautenschlager S., Brassey C.A., Button D.J., Barrett P.M. (2016). Decoupled form and function in disparate herbivorous dinosaur clades. Sci. Rep..

[bib32] Lautenschlager S., Gill P.G., Luo Z.X., Fagan M.J., Rayfield E.J. (2018). The role of miniaturization in the evolution of the mammalian jaw and middle ear. Nature.

[bib33] Lee S., Huang H.M., Lin C., Shih Y. (2000). In vivo and in vitro natural frequency analysis of periodontal conditions: an innovative method. J. Periodontol..

[bib34] Marinescu R., Daegling D.J., Rapoff A.J. (2005). Finite-element modeling of the anthropoid mandible: the effects of altered boundary conditions. Anat. Rec. A Discov. Mol. Cell. Evol. Biol..

[bib35] Maxwell E.E., Caldwell M.W., Lamoureux D.O., Budney L.A. (2011). Histology of tooth attachment tissues and plicidentine in *Varanus* (Reptilia: Squamata), and a discussion of the evolution of amniote tooth attachment. J. Morphol..

[bib36] McCurry M.R., Evans A.R., McHenry C.R. (2015). The sensitivity of biological finite element models to the resolution of surface geometry: a case study of crocodilian crania. PeerJ.

[bib37] Miedema F., Spiekman S.N.F., Fernandez V., Reumer J.W.F., Scheyer T.M. (2020). Cranial morphology of thetanystropheid *Macrocnemus bassanii* unveiled using synchrotron microtomography. Sci. Rep..

[bib38] Moazen M., Curtis N., O’Higgins P., Evans S.E., Fagan M.J. (2009). Biomechanical assessment of evolutionary changes in the lepidosaurian skull. Proc. Natl. Acad. Sci. U S A.

[bib39] Moreno K., Wroe S., Clausen P., McHenry C., D’Amore D.C., Rayfield E.J., Cunningham E. (2008). Cranial performance in the Komodo dragon (*Varanus komodoensis*) as revealed by high-resolution 3-D finite element analysis. J. Anat..

[bib40] Olejniczak A.J., Tafforeau P., Feeney R.N., Martin L.B. (2008). Three dimensional primate molar enamel thickness. J. Hum. Evol..

[bib41] Porro L.B., Holliday C.M., Anapol F., Ontiveros L.C.L.T., Ontiveros L.C.L.T., Ross C.F. (2011). Free body analysis, beam mechanics, and finite element modeling of the mandible of *Alligator mississippiensis*. J. Morphol..

[bib42] Rahman I.A., Lautenschlager S. (2016). Applications of three-dimensional box modeling to paleontological functional analysis. Paleontological Soc. Pap..

[bib43] Rayfield E.J. (2007). Finite element analysis and understanding the biomechanics and evolution of living and fossil organisms. Annu. Rev. Earth Planet. Sci..

[bib44] Rees J.S., Hammadeh M. (2004). Undermining of enamel as a mechanism of abfraction lesion formation: a finite element study. Eur. J. Oral Sci..

[bib45] Reichel M. (2010). A model for the bite mechanics in the herbivorous dinosaur *Stegosaurus* (Ornithischia, Stegosauridae). Swiss J. Geosciences.

[bib46] Renesto S., Avanzini M. (2002). Skin remains in a juvenile *Macrocnemus bassanii* nopcsa (Reptilia, Prolacertiformes) from the middle triassic of Northern Italy. Neues Jahrb. Geol. Palaontol. Abh..

[bib47] Rieppel O., Labhardt L. (1979). Mandibular mechanics in *Varanus niloticus* (Reptilia: Lacertilia). Herpetologica.

[bib48] Rieppel O., Scheffold B. (2019).

[bib49] Saint-Venant B.D. (1855). Mémoire sur la torsion des prismes. Mémoires des Savants étrangers.

[bib50] Sander P.M. (1999). The microstructure of the reptilian tooth enamel: terminology, function and phylogeny. Müncher Geowissenschaftliche Abhandlungen.

[bib51] Sandorff P.E. (1980). Saint-Venant effects in an orthotropic beam. J. Compos. Mater..

[bib52] Sellers W.I., Pond S.B., Brassey C.A., Manning P.L., Bates K.T. (2017). Investigating the running abilities of *Tyrannosaurus rex* using stress constrained multibody dynamic analysis. PeerJ.

[bib53] Sellers K.C., Schmiegelow A.B., Holliday C.M. (2019). The significance of enamel thickness in the teeth of *Alligator mississippiensis* and its diversity among crocodyliforms. J. Zool..

[bib54] Skinner M.M., Gunz P., Wood B.A., Hublin J.J. (2008). Enamel-dentine junction (EDJ) morphology distinguishes the lower molars of *Australopithecus africanus* and *Paranthropus robustus*. J. Hum. Evol..

[bib55] Taylor A.C., Lautenschlager S., Qi Z., Rayfield E.J. (2017). Biomechanical evaluation of different musculoskeletal arrangements in *Psittacosaurus* and implications for cranial function. Anat. Rec..

[bib56] Thomason J.J. (1991). Cranial strength in relation to estimated biting forces in some mammals. Can. J. Zool..

[bib57] Toupin R.A. (1965). Saint-Venant's principle. Archive Rational Mech. Analysis.

[bib58] Tseng Z.J., Mcnitt-Gray J.L., Flashner H., Wang X., Enciso R. (2011). Model sensitivity and use of the comparative finite element method in mammalian jaw mechanics: mandible performance in the gray wolf. PLoS One.

[bib64] Wang Q., Smith A.L., Strait D.S., Wright B.W., Richmond B.G., Grosse I.R., Byron C.D., Zapata U. (2010). The global impact of sutures assessed in a finite element model of a macaque cranium. Anat Rec.

[bib59] Wilken A.T., Middleton K.M., Sellers K.C., Cost I.N., Holliday C.M. (2019). The roles of joint tissues and jaw muscles in palatal biomechanics of the savannah monitor (*Varanus exanthematicus*) and their significance for cranial kinesis. J. Exp. Biol..

[bib60] Wilken A.T., Sellers K.C., Cost I.N., Rozin R.E., Middleton K.M., Holliday C.M. (2020). Connecting the chondrocranium: biomechanics of the suspensorium in reptiles. Vertebr. Zool..

[bib61] Wood S.A., Strait D.S., Dumont E.R., Ross C.F., Grosse I.R. (2011). The effects of modeling simplifications on craniofacial finite element models: the alveoli (tooth sockets) and periodontal ligaments. J. Biomech..

[bib62] Zapata U., Metzger K., Wang Q., Elsey R.M., Ross C.F., Dechow P.C. (2010). Material properties of mandibular cortical bone in the American alligator, *Alligator mississippiensis*. Bone.

