# Supplementary material for: Modeling tooth enamel in FEA comparisons of skulls: Comparing common simplifications with biologically realistic models
Source: iScience. 2021 Sep 28;24(11):103182. doi: 10.1016/j.isci.2021.103182 (PMC8567004; doi:10.1016/j.isci.2021.103182)
Supplement: Document S1. Figure S1 and Tables S1–S4 [file mmc1.pdf]

iScience, Volume *24*

Supplemental information

Modeling tooth enamel in FEA comparisons of skulls: Comparing common simplifications with biologically realistic models

Eva C. Herbst, Stephan Lautenschlager, Dylan Bastiaans, Feiko Miedema, and Torsten M. Scheyer

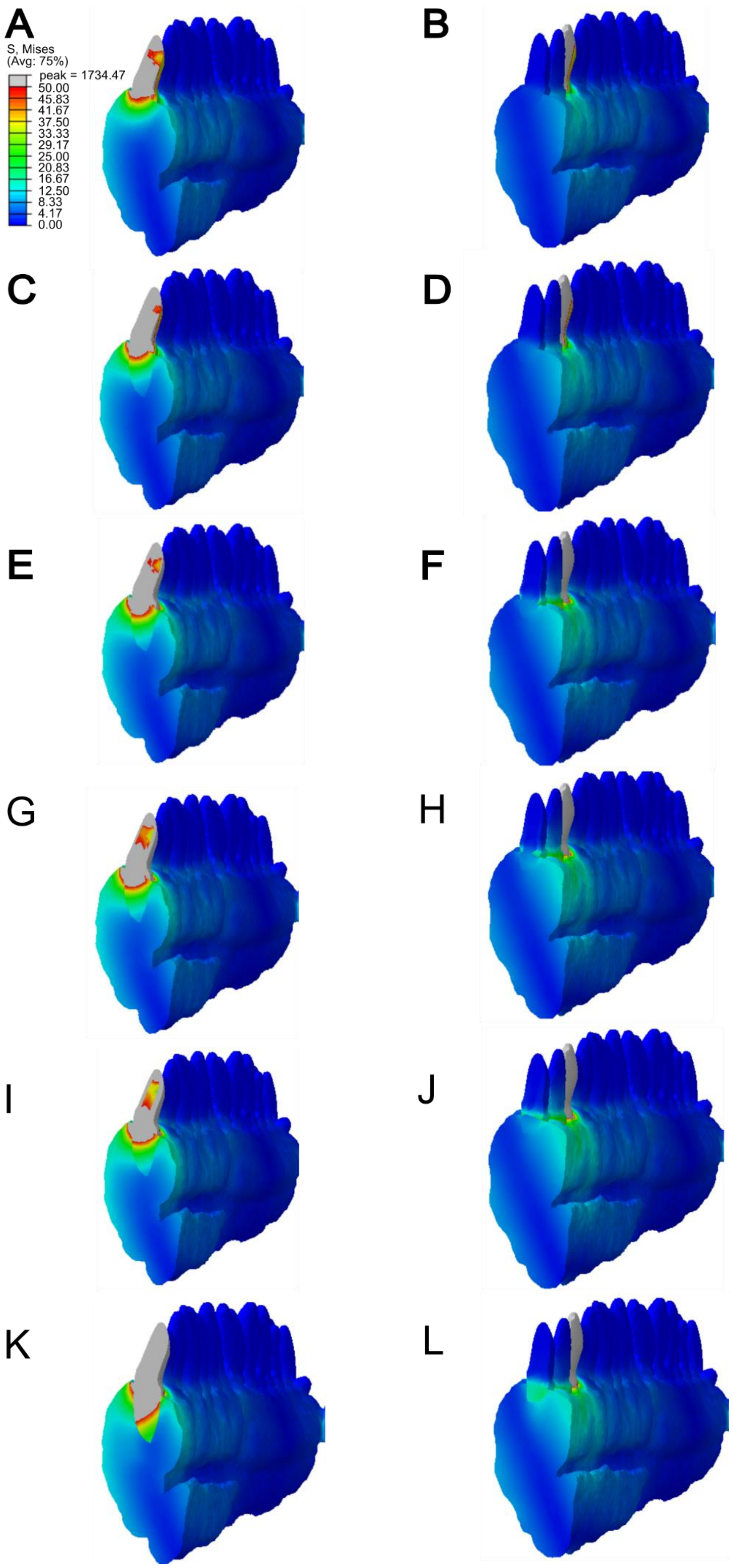
A
S, Mises
(Avg: 75%)
peak = 1734.47
50.00
45.83
41.67
37.50
33.33
29.17
25.00
20.83
16.67
12.50
8.33
4.17
0.00
B
C
D
E
F
G
H
I
J
K
L

Figure S1, related to Figure 4. Local stress distributions near the constrained tooth in *Macrocnemus*. A,C,E,G,I,K) Cross sections through the constrained tooth; B,D,F,H,J,L) Cross sections through the third tooth posterior to the constrained tooth. A,B) all bone model; C,D) all dentine; E,F) ~5% enamel; G,H) ~20% enamel; I,J) ~30% enamel; K,L) all enamel.

Table S1, related to Figures 2 and 3 and Table 1. Bite force (N) measurements at middle right tooth.

Specimen	*Macrocnemus*	*Erlikosaurus*	*Varanus*
Node #:	8543	679653	110725
all bone	1.59	101.52	2.44
all dentine	1.82	102.87	2.48
~5% enamel	2.22	105.22	2.57
~20% enamel	2.40	107.16	2.69
~30% enamel	2.48	108.04	2.76
all enamel	2.69	109.07	2.84

Table S2, related to Figures 5. Von Mises stress (mPA) at specific nodes for the 6 different tooth models for *Macrocnemus*.

	Anterior											Posterior
Node #:	245996	249316	256610	262155	266526	272854	278705	284414	293939	304956	313896	317462	319839
all bone	2.82339	1.34468	6.32696	8.82763	3.66126	3.53819	6.03173	5.49455	9.59346	8.43271	4.36189	7.5174	0.73867
all dentine	2.8477	1.13609	6.13746	9.14705	3.75113	3.58793	6.07759	5.40492	9.70256	8.4524	4.35821	7.40094	0.75764
~5% enamel	2.83017	0.94725	5.78946	9.579	3.93589	3.72618	6.18436	5.34105	9.89141	8.46186	4.33523	7.25789	0.79508
~20% enamel	2.81685	0.87393	5.63793	9.76109	4.02171	3.78766	6.22952	5.34903	9.97975	8.46645	4.32922	7.19595	0.81251
~30% enamel	2.81561	0.83801	5.56958	9.84986	4.06639	3.81536	6.24928	5.36111	10.0227	8.46953	4.32773	7.16545	0.82123
all enamel	2.70946	0.65128	5.4318	10.2298	4.31556	3.81558	6.23175	5.41733	10.1141	8.49353	4.3386	7.06153	0.84331

Table S3, related to Figure 5. Von Mises stress (mPA) at specific nodes for the 6 different tooth models for *Erlikosaurus*.

	Anterior												Posterior
Node #:	1095526	1094065	1092221	1092017	1133744	1096166	1019673	1099618	1155456	1101016	1101531	1120358	1138818
all bone	2.7143	2.9078	2.4317	3.8907	3.6674	5.0142	4.0427	4.4384	9.7425	3.0384	2.6672	2.5934	1.7352
all dentine	2.6496	2.8988	2.4215	3.7951	3.6292	4.9784	4.0340	4.4690	9.8154	3.0491	2.6683	2.6107	1.7260
~5% enamel	2.6571	2.9215	2.4421	3.7059	3.5816	4.9528	4.0316	4.5143	9.9229	3.0653	2.6662	2.6272	1.7279
~20% enamel	2.6513	2.9162	2.4645	3.6636	3.5669	4.9503	4.0396	4.5480	9.9983	3.0699	2.6594	2.6352	1.7286
~30% enamel	2.6502	2.9153	2.4755	3.6427	3.5592	4.9478	4.0425	4.5615	10.0286	3.0719	2.6569	2.6385	1.7293
all enamel	2.4355	2.8745	2.4318	3.5038	3.5202	4.8774	4.0215	4.5702	10.0529	3.0760	2.6655	2.6562	1.7235

Table S4, related to Figure 5. Von Mises stress (mPA) at specific nodes for the 6 different tooth models for *Varanus*.

	Anterior												Posterior
Node #:	175490	168604	169525	176403	172695	177518	177281	176272	159577	176798	155801	171809	166084
all bone	0.3556	0.2390	0.2133	0.5719	0.5151	0.0826	0.2042	0.2526	0.3375	1.0690	0.6671	0.0252	0.0002
all dentine	0.3171	0.2194	0.1387	0.5789	0.4963	0.0849	0.2046	0.2452	0.3313	1.0550	0.6521	0.0264	0.0002
~5% enamel	0.2625	0.1996	0.0831	0.5858	0.4775	0.0873	0.2040	0.2368	0.3251	1.0410	0.6362	0.0276	0.0002
~20% enamel	0.2493	0.1854	0.1156	0.5912	0.4624	0.0893	0.2042	0.2292	0.3202	1.0280	0.6212	0.0287	0.0002
~30% enamel	0.2395	0.1810	0.1501	0.5933	0.4560	0.0900	0.2036	0.2255	0.3181	1.0230	0.6146	0.0291	0.0002
all enamel	0.2316	0.1766	0.2144	0.5939	0.4489	0.0910	0.2031	0.2208	0.3153	1.0160	0.6057	0.0297	0.0002
